# A Silent Threat Unveiled: Invasive Fungal Sinusitis in a High-Risk Hematologic Malignancy Patient

**DOI:** 10.7759/cureus.61232

**Published:** 2024-05-28

**Authors:** Elexis B Price, Shresttha Dubey, Zoheb I Sulaiman, Hasan Samra, Gina Askar

**Affiliations:** 1 Infectious Disease, Medical College of Georgia at Augusta University, Augusta, USA; 2 Infectious Disease, Medical College of Georgia at WellStar MCG Health, Augusta, USA; 3 Pathology, Medical College of Georgia at WellStar MCG Health, Augusta, USA

**Keywords:** aspergillosis, opportunistic infections, hematologic malignancy, immunocompromised, invasive fungal sinusitis

## Abstract

Invasive fungal sinusitis (IFS) poses a fatal threat to patients with hematological malignancies or a history of allogeneic hematopoietic stem cell transplant (HSCT). While invasive aspergillosis, a subtype of IFS, remains rare in immunocompetent individuals, allogeneic HSCT recipients face a notable surge in incidence. Despite the rapid onset and progression of IFS, its clinical presentation is subtle, contributing to heightened mortality rates. Prompt surgical debridement and systemic antifungal therapy are required to yield positive results. Examining IFS cases in HSCT recipients is vital, providing insights into its clinical course, prevention strategies, and improved evaluation.

We present a rare presentation of IFS with *Aspergillus niger* in a relapsed acute myeloid leukemia patient post-HSCT. Two weeks after chemotherapy, the patient developed headaches and blood-tinged sinus drainage in the setting of pancytopenia. Radiologic and pathological findings confirmed the diagnosis of IFS, necessitating weeks of intensive anti-fungal therapy. Despite the initial positive response, the disease ultimately progressed to a fatal outcome. This case emphasizes that early detection is required for a favorable treatment response. Furthermore, it underscores the importance of heightened clinical suspicion, risk stratification, multidisciplinary care, and ongoing research for optimal management of IFS in allogeneic HSCT recipients.

## Introduction

In the realm of high-risk immunocompromised individuals, notably those with hematological malignancies, invasive fungal sinusitis (IFS) continues to pose a grave and potentially fatal threat, especially amidst profound neutropenia [1]. A myriad of causative organisms exists, with prominent fungal species including *Aspergillus* and *Mucorales* [2,3]. Particularly, *Aspergillus* species exhibit a heightened prevalence among patients with hematologic malignancy, with the risk attributed to immune system dysfunction compounded by cytotoxic chemotherapy-induced immunosuppression [4,5]. The implementation of preventative measures against graft-versus-host disease (GvHD) through immunosuppressive regimens further heightens the risk of individuals undergoing allogeneic stem cell transplants for oncologic treatment [5,6]. Despite the rapid onset and progression of IFS, its clinical presentation is often subtle and insidious resulting in a high mortality rate of approximately 50% [7,8]. Often, initial symptoms include fever, nasal congestion, and rhinorrhea resembling signs of a bacterial or viral upper respiratory tract infection, resulting in frequent misdiagnosis [5,9]. The fungi can rapidly invade the nasal mucosa and sinus into the orbit, cavernous sinus, or intracranial space causing more characteristic symptoms in extensive disease, including neurologic deficits, diplopia, and proptosis [4,10]. Due to this, heightened clinical suspicion in high-risk patients is necessary for timely diagnosis, a critical prognostic component [7]. Physical examination of patients with suspected IFS should include a comprehensive head and neck examination, ophthalmologic evaluation, and neurologic assessment of all cranial nerves [4].

Functional endoscopic sinus surgery (FESS) is a minimally invasive technique that employs radiological imaging as a navigational aid to evaluate and restore normal sinus function. FESS frequently serves as the initial diagnostic step revealing pale or ischemic mucosa, a hallmark finding in early IFS [9]. Mucosal transformations become evident as the disease advances, evolving from a pale and edematous mucosa into dark necrotic tissue and culminating in the formation of a thick eschar through a combination of ulceration and shedding [10]. Histopathologic assessment of tissue specimens is imperative, and the utilization of intraoperative frozen section analysis ensures timely confirmation of the presence of fungal organisms [4]. However, the effectiveness of cultures for identifying fungi is constrained due to the slow or non-existent growth of molds [5]. Although data on their performance is limited, laboratory testing for fungal biomarkers, which detect differing fungal cell wall components, can be used to supplement clinical evidence to diagnose IFS [5]. (1→3)-β-D-Glucan (Fungitell) and Galactomannan assays are among the more frequently used, with the latter particularly useful for detecting *Aspergillus* species [5]. When employing this strategy, it is critical to note that a negative result does not definitively exclude a fungal infection [5]. Distinct radiologic features apparent on computed tomography (CT) scans, such as mucosal thickening, also assist in facilitating the diagnosis of IFS [11]. The utilization of imaging is also essential for assessing fungal invasion, often necessitating magnetic resonance imaging (MRI) with contrast enhancement to provide more sensitive information, such as extranasal invasion or orbital extension [7]. Current treatment protocols encompass a multidisciplinary approach, involving surgical debridement, systemic antifungal therapy, and recovery of underlying immunodeficiency.

Prompt initiation of empiric antifungal therapy featuring liposomal amphotericin B (LAmB), selected for its broad antifungal efficacy and penetration of the central nervous system, is crucial. Any delay in therapy of ≥six days is associated with a twofold increase in mortality [10,12]. Intravenous therapy is recommended until a clear clinical response is achieved, typically requiring weeks of treatment, accompanied by close patient monitoring due to the significant side effect profile. Isavuconazole is a newly approved treatment for adults with invasive aspergillosis or mucormycosis that should be considered a step-down oral option or as first-line therapy in patients intolerant to amphotericin B [13]. In its entirety, tailoring antifungal therapy requires careful consideration of individual patient characteristics, with the essential involvement of infectious disease (ID) specialists for expert guidance.

Rare invasive fungal sinusitis in neutropenic individuals poses a unique challenge with advanced morbidity and high mortality. The multifaceted nature of IFS emphasizes the need for heightened clinical suspicion, a collaborative multidisciplinary approach, and prompt intervention. The case report presented below details a patient with relapsed acute myeloid leukemia (AML) post-transplant who was diagnosed with invasive *Aspergillus* sinusitis. Although the condition initially responded to complex medical and surgical management, it eventually advanced, involving orbital invasion and ultimately resulting in an unfortunate fatal outcome.

## Case presentation

A 53-year-old male, with a history of relapsed AML status post-allogeneic hematopoietic stem cell transplant (HSCT) in December 2020 (CMV D+/R+) complicated by GvHD, presented to an outside hospital in September 2023 with persistent high fevers, headaches, and mild blood-tinged sinus drainage two weeks after completing inpatient chemotherapy with methotrexate and decitabine. Following transfer to our hospital for a higher level of care, the patient was hemodynamically stable on admission with mild fevers (up to 38.6°C) and endorsed complaints of frontal lobe headaches and blood-tinged nasal secretions.

**Figure 1:**
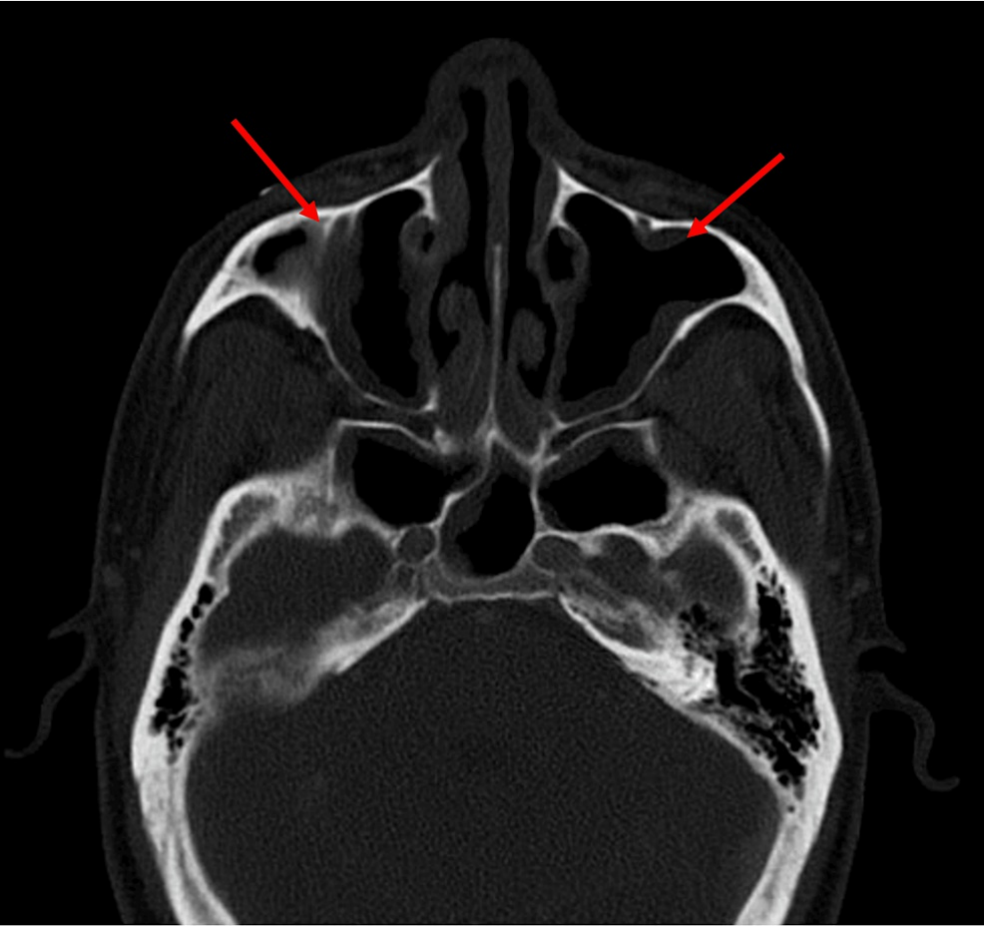
CT sinuses with IV contrast. The radiographic image shows moderate mucosal thickening of bilateral maxillary sinuses with partial obstruction of left > right maxillary ostia (red arrows). CT: computed tomography

Labs were significant for pancytopenia with white blood cell (WBC) of 400/mm^3^, hemoglobin of 11.2 g/dL, and platelets of 30,000/mm^3^ (Table 1). Physical examination was primarily unremarkable. In conjunction with his standard prophylaxis (acyclovir, fluconazole, and trimethoprim-sulfamethoxazole) and a reduction in immunosuppressive therapy, empiric antimicrobial coverage with cefepime was initiated for neutropenic fever. Concerns for IFS led to a CT scan of the sinuses, revealing worsening mucosal disease of the paranasal sinuses without bony erosions or disease extension (Figure 1).

**Table 1:** Laboratory parameters.

Laboratory parameter	Levels at presentation	Normal range
White blood cell	400/mm^3^	4,500–11,000/mm^3^
Hemoglobin	11.2 g/dL	14.0–18.0 g/dL
Platelet count	30,000/mm^3^	150,000–400,000/mm^3^

**Figure 2:**
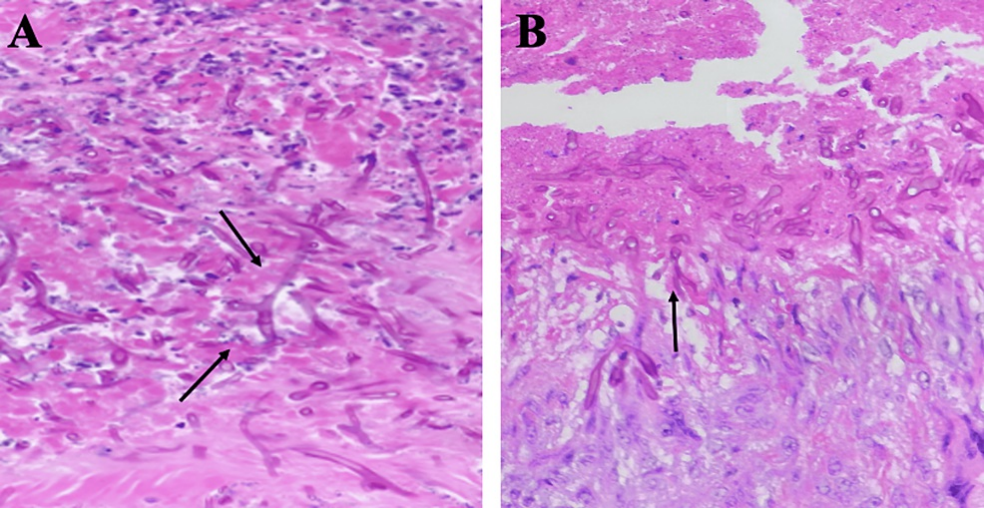
Frozen pathology. A and B show *Aspergillus* species are thinner and septate, with regular branching and branches at acute angles (black arrows) when compared with the organisms of mucormycosis (45° as opposed to 90°).

His antimicrobials were broadened from acyclovir to posaconazole and cefepime to piperacillin/tazobactam, and vancomycin was added. A bedside rigid nasal endoscopy with biopsy was performed by the otolaryngology service showing crusting of the right medial meatus with pale mucosa. Frozen pathology returned positive for invasive fungal elements concerning for mucormycosis (Figures 2A, 2B).

The ID service was consulted for antifungal recommendations. Posaconazole was discontinued and the patient was started on dual mold therapy with intravenous LAmB and Cresemba (isavuconazonium sulfate). Serum Fungitell was ordered and returned with a value of 42 pg/mL. Subsequent nasal endoscopy with debridement and bilateral FESS revealed *Aspergillus niger* in sinus tissue cultures; however, his initial histopathology showed concerns for two different fungal elements consistent with *Aspergillus* and *Mucor*. Serum *Aspergillus* antigen returned <0.500. Complex pharmaceutical management of antimicrobial agents was modified throughout the patient’s hospitalization based upon negative cultures, excluding one blood culture resulting in a presumed contaminant of *Paenibacillus*.

In the following days, the patient reported ongoing visual floaters but experienced an improvement in subjective fever and chills. Five days later, remaining on systemic antifungal therapy, the patient underwent a second debridement showing no further invasive fungal elements. Subsequent MRI of the face, neck, and orbits also confirmed no evidence of intracranial extension. Due to clinical improvement, the patient was discharged on outpatient Cresemba treatment. Unfortunately, the patient returned a few weeks later in October 2023 for complaints of neutropenic fevers up to 40.6°C, congestion, and right orbital edema. Repeat CT of the sinuses showed interval worsening of diffuse paranasal sinus inflammatory changes and hyperdense components of multiple sinuses concerning for IFS. The patient was continued on Cresemba and restarted on LAmB therapy. He underwent FESS with ENT which revealed crusting with fungal debris requiring extensive regional removal, and intraoperative tissue cultures grew *Aspergillus niger* again. The patient endorsed ongoing diplopia, floaters, and painful eye movement. Right eye proptosis and periorbital edema were observed on examination. While MRI of the face, neck, and orbits suggested no intracranial extension, interval extension into the right orbit and extraocular muscles was observed, requiring retrobulbar amphotericin B injections. Following treatment, the patient experienced visual symptomatic improvement but remained critically ill with persistent neutropenia. Due to ongoing neutropenia and worsening IFS, the patient and family elected to transition to home hospice.

## Discussion

This case report highlights a rare presentation of IFS due to *Aspergillus niger* in an immunocompromised adult. The patient’s hematologic malignancy, particularly relapsed AML and a history of HSCT complicated by GvHD are well-established risk factors for IFS [14,15]. Given the aggressive spread and poor prognosis observed in this patient, this case aligns with existing literature supporting the association between hematologic malignancy treated with HSCT and IFS. Thus, the occurrence of febrile neutropenia and vague rhino-nasal symptoms in this high-risk patient population underscores the critical need for immediate evaluation, ensuring drastic management strategies can be taken promptly if needed.

The occurrence of invasive aspergillosis (IA) presents a significant contrast, with exceedingly rare incidence in immunocompetent individuals. However, the occurrence of IA among HCST recipients varies, depending on the classification of the transplant. Although based on limited data, in allogeneic HSCT, the incidence of IA is estimated at 6.7%, whereas in autologous HSCT, it is approximately 0.4% [15]. The risk of IA in HSCT recipients is heightened by acute or chronic GvHD involving treatment, active underlying hematologic malignancy, and environmental exposures such as proximity to construction work, gardening, and marijuana usage [16,17]. Furthermore, among allogeneic HSCT recipients, individuals requiring reinduction chemotherapy due to relapse during the late post-engraftment period, as exemplified by our patient, are at a heightened risk of IFS. Unfortunately, the patient in this case was particularly vulnerable to IA due to numerous risk factors, underscoring the necessity of thorough risk stratification among HSCT recipients. Clear documentation of these factors is essential for easy identification, ensuring healthcare personnel remain vigilant for IFS, particularly IA, when encountering such patients. Physicians should also thoroughly counsel high-risk individuals about the subtle indications of IFS and risk-modifying behaviors.

Antifungal prophylaxis in HSCT recipients, commonly fluconazole, has reduced the incidence of IFS, especially due to *Candida* spp. Overall, data regarding the effectiveness of mold-active prophylaxis within this patient population is limited; however, a recent meta-analysis compromising 20 randomized controlled trials revealed compelling evidence [18]. Mold-active prophylaxis demonstrated a reduction in invasive fungal infections (IFIs) (p = 0.003), a decrease in the risk of IA (p = 0.0004), and a lowering of IFI-related mortality (p = 0.03) when compared to fluconazole prophylaxis. It is important to note, however, that mold-active prophylaxis was associated with an increased risk of adverse events leading to antifungal discontinuation (p = 0.004). Nonetheless, the consideration of mold-active prophylaxis, such as posaconazole or voriconazole, is warranted for patients with a history of allogeneic HSCT who are deemed at high risk for IA [16,19]. While data on the prophylactic use of isavuconazole is limited, it may be considered for patients at risk of drug-drug interactions or QT prolongation [16].

Researchers have worked on an algorithm to improve outcomes regarding IFS among children with hematologic malignancies and/or a history of stem cell transplants [20]. The researchers reported an 18.5% reduction in mortality after implementing their protocol in a sample size of 33 cases (p = 0.29). Although the results were not statistically significant, the outcomes and small sample size warrant further investigation into how the evaluation of IFS can be improved in these patient populations. An important observation from the algorithm is that ENT consults for more invasive diagnostic procedures (endoscopy and biopsy) were started after five days of febrile neutropenia in patients. Chen et al. also found higher mortality rates of IFS in acute leukemia patients with greater than 10 days of febrile neutropenia [14]. Of the 33 patients studied, 25 were diagnosed via endoscopy, even though 17 reported no rhino-nasal complaints. This observation, in combination with ENT evaluation triggered after five days of neutropenia, may suggest that early and aggressive evaluation of febrile neutropenia can improve outcomes in patients such as the one presented here. As described in the Introduction, the clinical presentation is often subtle and insidious which makes thorough and careful evaluation of invasive fungal infections crucial. Moreover, when clinical suspicion for IFS is heightened, an ENT evaluation is deemed a surgical emergency [5]. ENT specialists leverage radiological findings to direct macroscopic examination and tissue sampling, facilitating expedited pathologic review and enabling therapeutic management through FESS, which involves the debridement of necrotic tissue [5].

The destructive nature and rapid progression of IFS, as seen here, warrants further investigation into appropriate evaluation and prevention. Insights into the limited but emerging data on mold-active prophylaxis and its associated risks provide valuable considerations for clinicians managing these high-risk patients, and its usage may enhance outcomes in the future.

## Conclusions

This case report emphasizes the unique risks of IFS, specifically IA, in allogeneic HSCT recipients with a history of treated GvHD and prolonged neutropenia. The insidious nature of IFS highlights the imperative for heightened clinical suspicion among healthcare providers. Furthermore, the increased incidence among HSCT recipients, particularly allogeneic HSCT recipients, accentuates the importance of early risk stratification. While conflicting evidence exists, insights from emerging research on mold-active prophylaxis present potential avenues for optimizing patient outcomes in the future. However, continued data collection and ongoing endeavors toward novel therapies remain imperative to decrease mortality associated with IFS. Overall, this case underscores the critical importance of heightened clinical suspicion, a multidisciplinary approach, proactive management strategies, and ongoing research to enhance the understanding and treatment of IFS in the context of allogeneic HSCT recipients.
